# Comparative Study of Cytomorphological Robinson's Grading for Breast Carcinoma with Modified Bloom-Richardson Histopathological Grading

**DOI:** 10.1155/2013/146542

**Published:** 2013-09-25

**Authors:** Neelam Sood, Jitendra Singh Nigam, Poonam Yadav, Shivani Rewri, Ankit Sharma, Anita Omhare, Jaya Malhotra

**Affiliations:** Department of Pathology, D.D.U. Hospital, Harinagar, New Delhi 110066, India

## Abstract

*Objective*. To study the correlation of cytomorphological Robinson's grading for breast cancers with a modified Bloom-Richardson histopathological grading. *Materials and Methods*. One hundred sixteen cytologically malignant breast tumour cases were included in this study and correlated with paraffin embedded sections. Breast lumps were varied from less than 1 cm to 11 cm in greatest dimension. FNA was performed from different sites of the breast lump, and smears were stained with Giemsa and H&E stain and evaluated for cytological grading according to Robinson's grading system. Paraffin embedded tissue sections were stained with hematoxylin and eosin stain and graded according to modified Bloom-Richardson grading system. Comparison between these two grading systems was done. *Results*. Cytologically grade I, grade II, and grade III cases were 13.8%, 64.65%, and 21.55%, respectively. Histologically 25%, 54.31%, and 20.69% cases were grade I, grade II, and grade III, respectively. Concordance rate between cytology and histology of grade I, grade II, and grade III tumors was 75%, 70.67%, and 60% respectively. The absolute concordance rate was 68.97%. *Conclusion*. In the era of multiple treatment modalities and neoadjuvant therapy, cytological grading can be used as a prognostic factor for better management of patients.

## 1. Introduction

Breast cancer is the commonest cancer in urban Indian women and the second commonest cancer in the rural women next to cervical carcinoma. Due to general indifference towards the health of females in the Indian society, lack of an organized breast cancer screening program, and paucity of diagnostic aids, majority of breast cancers are diagnosed at a relatively advanced stage [1]. Since last decade, cytodiagnosis has gained importance due to rapid results at low cost and may help in early diagnosis in country like India. FNAB is a reliable method for the initial evaluation and diagnosis of palpable masses of the breast. In addition, it also has the ability of providing necessary prognostic/predictive information, particularly for patients that may undergo neoadjuvant therapy [2]. The standard prognostic factors, recognized by the National Cancer Institute in 1990, include lymph node status, tumor size, nuclear grade, steroid receptor content, tumor type, and cellular proliferation rate and recommend that for patients who undergo preoperative chemotherapy or radiotherapy, breast fine-needle aspirates can be used to provide prognostic information [3]. The grading of breast cancer on fine-needle aspiration help in understanding of the biology of the disease, to predict the outcome, select the appropriate treatment modality, explain variations in treatment outcome, plan specific therapeutic interventions, and, occasionally, alleviate patient anxiety [3]. Elston's modified Bloom and Richardson method is a widely accepted tumor grading system and has been found to have good prognostic correlations [4]. Histological grade forms part of the multifactorial Nottingham prognostic index, together with tumour size and lymph node stage and used to stratify individual patients for appropriate therapy [4]. Various studies correlate the cytological grading with Elston's modified Bloom and Richardson method. Robinson's cytological grading (RCG) to correlate with Elston's modified Bloom and Richardson method because of better concordance rate than other cytological grading systems. Also RCG has more objective set of criteria and easy reproducibility [5–7].

In present study, Robinson's cytological grading was done on cytology and compared with modified Bloom-Richardson system on paraffin embedded sections.

## 2. Materials and Methods

One hundred sixteen cytologically malignant breast tumour cases were included in this study and correlated with paraffin embedded sections. Breast lumps were varied from less than 1 cm to 11 cm in greatest dimension. 60 cases were in left breast, and 56 cases, in right breast with commonest age group were 56 to 60 years. 54 cases were premenopausal, and 62 cases were postmenopausal. FNA was performed from different sites of the breast lump using a 10 mL disposable syringe and 22/23-gauge needle without local anesthesia. FNA smears were stained with Giemsa and H&E stain and evaluated for cytological grading according to Robinson's grading system. Paraffin embedded tissue sections obtained from mastectomy specimens were stained with hematoxylin and eosin stain and graded according Elston-Ellis modification of Bloom-Richardson grading system. Comparison between these two grading systems was, done and concordance rates between each grade were calculated separately, and absolute concordance was calculated between all three corresponding grades. Kappa coefficient was used to compare the agreement for each grade.

## 3. Results

**Figure 1:**
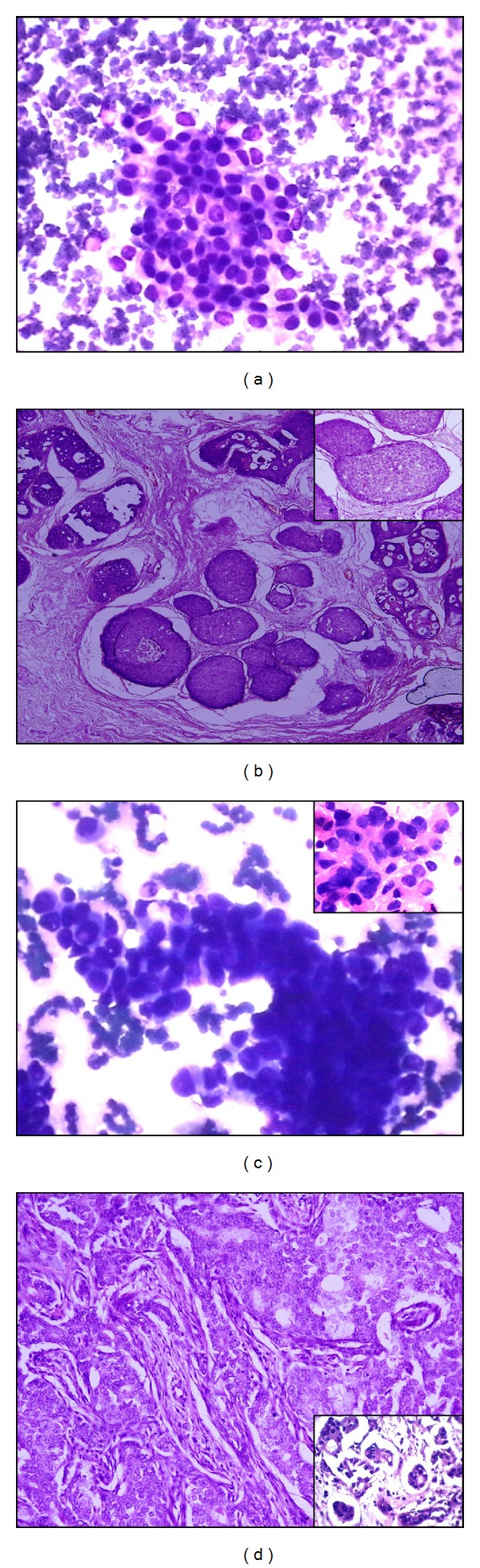
(a) Loosely cohesive cluster of mild pleomorphic ductal cells with smooth nuclear membrane and indistinct nucleoli—cytologic grade I (Giemsa ×400). (b) Invasive breast carcinoma with tubular/glandular differentiation, little increase in size, regular outlines, and uniform nuclear chromatin—histological grade I (H&E ×100, inset, H&E ×400). (c) Loosely cohesive cluster as well as single cells with moderate pleomorphism, slightly irregular nuclear membrane, and noticeable nucleoli. Nuclei are three to four times the erythrocytes—cytologic grade II (Giemsa ×400) (inset, H&E ×1000). (d) Invasive breast carcinoma with cords, islands with tubular differentiation, moderate variability in size and shape, open vesicular nuclei, and visible nucleoli—histological grade II (H&E ×100, inset, H&E ×400).

**Figure 2:**
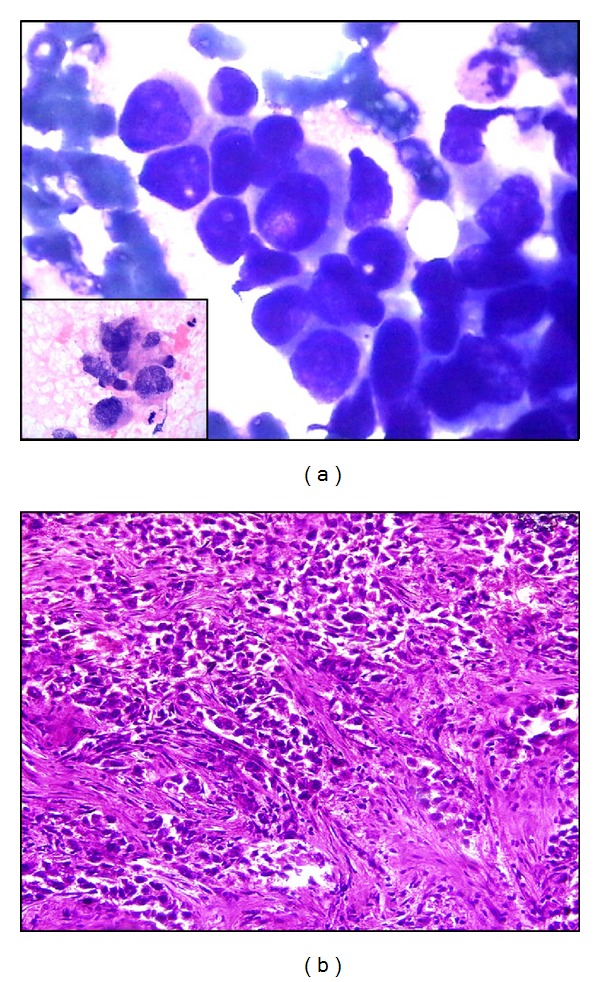
(a) Markedly pleomorphic ductal cells irregular nuclear margin and prominent nucleoli, coarse chromatin, and mitosis—cytologic grade III (Giemsa ×1000) (inset, H&E ×1000). (b) Invasive breast carcinoma infiltrative islands, with minimal tubular differentiation, marked variability in size and shape, open vesicular nuclei, visible nucleoli, and mitosis is seen histological grade III (H&E ×400).

All cases were graded cytologically and histologically using cytologic Robinson's grading system and modified bloom-Richardson histologic grading system, respectively. Comparison between both grading systems was done. The details of all cytological features are evaluated according to histological grading, and cytological grading is shown in Table 1. In this study, 13.8%, 64.65%, 21.55% cases were cytologically grade I (Figure 1(a)), grade II (Figure 1(c)), and grade III (Figure 2(a)) respectively. Histologically 25%, 54.31%, 20.69% cases were grade I (Figure 1(b)), grade II (Figure 1(d)), and grade III (Figure 2(b)), respectively. Concordance rate between grade I tumors in cytology and histology was 75%, for grade II tumors, it was 70.67%, and for grade III tumors it was 60%. The absolute concordance rate between all three corresponding grades was 68.97% (Table 2).

Discordance between cytological and histological grading was seen in 36 (31.03%) cases only. Most mismatching cases were seen in tumour size ranging from 2 cm to 5 cm (Table 3), and most the common age group was 36–60 years (58.33%) followed by 25–35 years (27.78%). 79 cases were of borderline cytological scoring, that is, scoring between 11 and 12 and between 14 and 15. Sixteen cases with discordance belonged to borderline cytological grades.

The kappa statistics was done to measure the strength of agreement between cytological and histologic grades. Kappa value for grade I tumors, grade II, and grade III tumors was 0.43, 0.43, and 0.51, respectively which indicates moderate agreement between cytological and histological grading systems.

The nuclear features of histology mirrored the cytological grading. Cytological grading of histology of grade I showed 22 cases of grade I, 6 cases of grade II, and one case of grade III. On cytological grading of histology grade II, cytological features of grade III were seen in 9 cases and 3 cases of cytological grade I. On cytological grading of histology grade III, cytological features of grade II were seen in 7 cases, and cytological features of grade I were seen in one case.

## 4. Discussion

A palpable breast lump is a common clinical problem that is presented to surgeons, gynaecologists, and general practitioners and a multidisciplinary approach based on the “triple test,” analyzing clinical and radiologic findings in conjunction with the pathologic features to diagnose the lesion and determine the best treatment plan for the patient [8]. Preoperative biopsy is also used for hormone receptor analysis and for the evaluation of other prognostic parameters by various ancillary techniques [8]. There are many cytologic grading systems for breast carcinoma, and they have good correlation with Elston-Ellis grading system. Because of more sensitivity, simplicity, more objective set of criteria and easy reproducibility, Robinson's method was considered better than the other methods [5–7, 9]. The concordance rate of histological and cytological grading was ranging from 56.9% to 89.1% [9]. In the present study, absolute concordance rate was 68.97% and observed predominance of grade II tumours, which is in corroboration with previous studies by Khan et al. [10], Das et al. [11], and Wani et al. [6, 12], Yu et al. correlated the Robinson's cytological grading with Bloom-Richardson's histopathological grading in 59 cases and observed substantial strength of agreement for grade I and grade II tumours with nearly perfect concordance in grade III tumours between cytology and histopathology [13]. In the present study, Highest of discordance was observed in grade III tumours followed by grade II and grade I. Discordance may be due to subjectivity when assessing cytological features that are not included in histological grading. Yu et al. observed discordance between cytological and histological grading due to difficulties in detecting mitoses or tubules in the cytology of breast carcinoma while nuclear features have contributed more to cytological grading which is one of the criteria in histopathological grading [14]. The extent of tubule formation, number of mitotic figures and degree of nuclear pleomorphism are the important factors of MBR histological grading systems but several features like nuclear margin, chromatin pattern and nucleoli that are included in cytological grading are not of much importance in histopathological grading [5]. Cangiarella and Simsir observed that cytological features, dissociation, cell size, cell uniformity, nucleoli, nuclear margin, and chromatin show a strong correlation with cytological grade and found that cell dissociation, cell uniformity, and nucleoli are the most influential features [7]. The features like cellular arrangement, degree of cellular pleomorphism, degree of nuclear pleomorphism, and absence of myoepithelial cells are important to diagnose carcinoma on histopathology [5]. The degree of cell dissociation indicates cell cohesion status, to an extent, and the degree of expression of the E-cadherin/catenin complex. Several studies showed that neoplasm with greater cell dissociation shows a higher incidence of regional lymph node metastasis [15–17]. Cytological grading before surgery is important because preoperative neoadjuvant chemotherapy is becoming common for the treatment of breast cancer and helps in the selection of appropriate regime [17]. The cytological prognostic grading system helps in identifying fast growing tumors (grade III), which are more likely to respond to chemotherapy than the low grade tumors; slow growing tumors may be better suited to pretreatment with Tamoxifen [18]. The high accuracy rate, rapid diagnosis, negligible risk of tumor spread, minimal subjective discomfort, insignificant complications, and utility for multiple lesions of FNAC; FNAC grading is compared with histology grading and is useful in assessing the tumor behaviour and prognosis and guiding neo adjuvant chemotherapy. Various attempts have been made to determine various prognostic parameters on FNA materials [9]. The National Cancer Institute (NCI), Bethesda, MD, USA, sponsored conference had also recommended that tumor grading on FNA material should be incorporated in FNA reports for prognostication [16].

## 5. Conclusion

 In the era of multiple treatment modalities and neoadjuvant therapy, cytological grading helps in evaluating the aggressiveness of tumour, neo adjuvant chemotherapy and can be used as a prognostic factor for better management of patients.

## Figures and Tables

**Table 1:** Cytological features on FNAC grading and histological grading.

	Cytological features on FNAC grading			Cytological features on histological grading		
	Grade I	Grade II	Grade III	Grade I	Grade II	Grade III
Cell dissociation						
Mostly clusters	2	2		2	18	
Single cells and clusters	12	71	20	21	44	15
Mostly single cells	2	2	5	6	1	9
Nuclear size						
1-2x RBC	5			7	10	2
3-4x RBC	11	50	3	21	48	5
5x or more RBC		25	22	1	5	17
Cell uniformity						
Monomorphic	9			8	10	2
Mildly pleomorphic	7	45		20	48	5
Pleomorphic		30	25	1	5	17
Nucleoli						
Indistinct/small	12	19		14	20	3
Noticeable	4	51	14	13	39	8
Abnormal		5	11	2	4	13
Nuclear margin						
Smooth	8			8	10	2
Slightly irregular/folds and grooves	8	67	19	20	50	9
Buds and clefts		8	6	1	3	13
Chromatin pattern						
Vesicular	9	3		10	12	2
Granular	7	70	20	19	48	7
Clumping and clearing		2	5		3	15
Total cases	16	75	25	29	63	24

**Table 2:** Comparison of Robinson's cytological grading with modified Bloom-Richardson grade.

	Histological grading				Concordance rate
		Grade I	Grade II	Grade III	
Cytological grading	Grade I	12	3	1	75%
	Grade II	14	53	8	70.67%
	Grade III	3	7	15	60%

	Absolute concordance rate				68.97%

**Table 3:** Cytological and histological grading with tumour size.

Tumour size	FNAC grading			Histological grade		
	Grade I	Grade II	Grade III	Grade I	Grade II	Grade III
1-2 cm	6	15	3	8	13	2
2–5 cm	8	42	14	16	38	9
5 cm or more	2	18	8	5	12	11
